# Quantification of cavitating flows with neutron imaging

**DOI:** 10.1038/s41598-024-76588-3

**Published:** 2024-11-06

**Authors:** I. K. Karathanassis, M. Heidari-Koochi, F. Koukouvinis, L. Weiss, P. Trtik, D. Spivey, M. Wensing, M. Gavaises

**Affiliations:** 1 School of Science & Technology, City, University of London, Northampton Square, London, EC1V 0HB UK; 2 Chair of Technical Thermodynamics, Friedrich-Alexander-Universität Erlangen-Nürnberg, Am Weichselgarten 8, 91058 Erlangen, Germany; 3 Laboratory for Neutron Scattering and Imaging, Paul Scherrer Institute (PSI), 5232 Villigen PSI, Switzerland; 4 Lubrizol European Research and Development Centre, Nether Lane, Hazelwood, Derby, DE56 4AN UK; 5 Present Address: School of Physics, Engineering and Computer Science, University of Hertfordshire, Hatfield, AL10 9AB UK; 6 Present Address: Department of Mechanical Engineering and Materials Science and Engineering, Cyprus University of Technology, Arch. Kyprianou 30, 3036 Limassol, Cyprus

**Keywords:** Irradiation imaging, Microfluidics, Two-phase flows, Phase change, Viscoelasticity, Fluid dynamics, Imaging techniques, Experimental nuclear physics, Mechanical engineering

## Abstract

The current experimental investigation demonstrates the capability of neutron imaging to quantify cavitation, in terms of vapour content, within an orifice of an abruptly constricting geometry. The morphology of different cavitation regimes setting in was properly visualised owing to the high spatial resolution of 16 μm achieved, given the extensive field of view of 12.9 × 12.9 mm^2^ offered by the imaging set-up. At a second step, the method was proven capable of highlighting subtle differences between fluids of different rheological properties. More specifically, a reference liquid was comparatively assessed against a counterpart additised with a Quaternary Ammonium Salt (QAS) agent, thus obtaining a viscoelastic behaviour. In accordance with previous studies, it was verified, yet in a quantifiable manner, that the presence of viscoelastic additives affects the overall cavitation topology by promoting the formation of more localised vortical cavities rather than cloud-like structures occupying a larger portion of the orifice core. To the authors’ best knowledge, the present work is the first to demonstrate that neutron imaging is suitable for quantifying in-nozzle cavitating flow at the micrometre level, consequently elucidating the distinct forms of vaporous structures that arise. The potential of incorporating neutron irradiation for the quantification of two-phase flows in metallic microfluidics devices has been established.

**Supplementary Information:**

The online version contains supplementary material available at 10.1038/s41598-024-76588-3.

## Introduction

Cavitation, as a flow phenomenon, is able to designate the effectiveness of devices and procedures relevant to numerous biomedical applications including heart valves^1^, needle-free injection devices^2^, ultrasound imaging devices^3^, bacteria removal^4^, and assisted drug delivery^5^, as well as a wide range of industrial applications including surface cleaning^6^, inkjet printers^7^, propellers^8^, fuel injectors^9^ and rocket engines^10–12^. The onset of vapour cavities, owing to the rapid liquid depressurisation beyond the fluid saturation pressure is accompanied by bubble dynamics and compressibility-related processes, e.g., propagation and eventual collapse when higher pressures are realised. Shockwave formation is manifested at length and time scales of the order of microns and microseconds, respectively. Cavitation evolution and collapse, if properly controlled, can be of technological importance for matter fragmentation and spray atomisation, while, on the contrary, as a stochastic process it leads to fluctuating device performance, noise, vibrations and material erosion^13–18^.

Cavitation regimes associated with coherent recirculation patterns in wall-bounded flows can be broadly classified as cloud and vortical cavitation. Cloud cavitation emerges in regions of flow separation due to the low pressure prevailing in the core of cross-flow vortices. It commonly arises with an unsteady trailing edge from which vortices, eventually obtaining a horseshoe form, are detached at characteristic frequencies. Elongated vortical cavities emanate from coherent vortex roll-up in regions away from wall boundaries and are associated with inherent three-dimensional flow instabilities. Cavitation within internal flow layouts is primarily visualised with the use of optical imaging^19–21^. This can be made possible only through the fabrication of transparent replicas of the actual configurations of interest^22–25^. However, as the overall dimensions of a flow device are reduced and the realm of microfluidics is approached, limitations with respect to optical access, test-piece rigidity and spatial resolution of the imaging system come into play.

X-ray imaging, especially using high-flux synchrotron radiation, has gained momentum recently as a technique capable of visualising cavitating flows in internal geometries^26^, however the relevant literature is still scarce, as few facilities globally offer the high-end infrastructure required. X-ray images, or radiographies are the result of either amplitude reduction of the X-ray wave or a shift in its phase. The corresponding imaging techniques are commonly referred to as X-ray absorption and Phase Contrast Imaging (XPCI), respectively. XPCI offers increased signal-to-noise ratio, compared to pure absorption imaging, enabling time-resolved measurements. However, XPCI is performed utilising a, so called, white beam, i.e., comprising a spectrum of X-ray energies. This results in beam ‘hardening’ in the sense that the average energy of the X-ray beam increases as it penetrates deeper into the material, since lower-energy X-ray photons are absorbed at smaller depths compared to higher-energy photons^26^. This leads to a variable absorption coefficient rendering the derivation of mean vapour mean-path length unfeasible. Recently, Tekawade et al.^27^ performed XPCI tomographic reconstruction of the cavitating flow forming inside a steel injector at realistic operating conditions. It is important to emphasise that typical diameters of injection nozzles are of the order of 100–200 μm, while injection pressures for diesel fuel exceed 200 MPa. In a previous study of the authors’ group, time-resolved XPCI was employed to illustrate the transient formation and collapse of string-like cavitation in a carbon-fibre orifice with a flow constriction to a minimum diameter of 1.5 mm^28^. In a follow-up experiment, the influence of non-Newtonian flow behaviour and more specifically viscoelasticity on cloud and vortical cavitation was scrutinised in the same orifice layout^29^. A recent investigation by Soyama et al.^30^ employing ultra high-speed imaging at 1.1 million frames per second conducted at the fourth-generation source of the European X-ray Free Electron Laser, verified through XPCI the topology and dynamics of elongated coherent vortical cavities initially shown in^28^.

As already mentioned, cavitation quantification, i.e., the determination of the actual volume of generated vapour, in opaque objects challenges the limits of modern non-intrusive visualisation techniques. Mitroglou et al.^31^, with the use of a commercial cone-beam X-ray source, performed computed tomography to quantify the three-dimensional cavitation cloud arising within a millimetre-sized nozzle made from Polyether Ether Ketone (PEEK). Duke et al.^32^ have performed quantitative experiments on a beryllium orifice using synchrotron radiation. A raster scanning method was realised to irradiate the internal diameter of 500 μm with a beam spot size of 5 × 6  µm^2^. The density distribution of the prevailing sheet cavity was obtained, however, the working medium was doped with cerium as a contrast-enhancing agent. Very recently, Karathanassis et al.^33^ resolved the transient cavitation structures forming in a throttle orifice at 67,890 frames per second taking advantage of the high-flux pink beam produced by a helical superconducting undulator. Both cloud and vortical cavitation were quantified along with distinct transient features, such as vortex shedding.

A monochromatic high flux beam from a synchrotron source, necessary for flow quantification, can offer spatial and temporal resolutions of the order of micrometres and microseconds. Nevertheless, X-rays suffer from extensive attenuation during interaction with matter and are therefore inherently limited to low signal-to-noise ratios when penetrating dense materials. Besides, resolution deteriorates if the field of view is larger than a few millimetres. On the contrary, neutrons interact with matter less strongly than X-ray photons and can penetrate thick metals such as titanium and lead. Neutrons tend to be attenuated mostly by light elements, such as hydrogen or carbon^34^ characterised by high absorption cross-section values, while X-rays, are attenuated in dense materials with high atomic numbers due to photoelectric absorption through interactions with atomic electrons. Neutron imaging is applicable in a vast range of research fields such as material science^35^, plant^36^ and insect physiology^37^, archaeology^38^, among others.

Emphasising on fluid flow processes, neutron imaging has been employed for the visualisation of gas structures in two-phase flows^39–41^ and the determination of unsteady two-phase velocity fields^42^, as well as for the measurement of component concentration variation in vaporising droplets^43^ or complex liquid mixtures^44^. With respect to flows realised in industrial applications^45^, neutron irradiation has been employed for the visualisation of the two-phase mixture forming in fuel cells or electrolysers^39^, porous media^40^, also with emphasis on soil physics^46^, heat pipes^47^ and packed bed reactors^48^. Especially referring to fuel cells and electrolysers, neutron imaging constitutes an ideal imaging technique owing to its capability to penetrate material layers and high hydrogen sensitivity. Cochet et al.^49^ utilised neutron irradiation to visualise the two-phase mixture forming in a novel, patterned gas diffusion layer facilitating evaporative cooling of a polymer electrolyte fuel cell with a spatial resolution of 100 μm. Iranzo et al.^50^ visualised the water distribution at the bipolar plates of a proton exchange membrane (PEM) fuel cell with an effective resolution of 300 μm. Selamet et al.^51^ illustrated the gas bubble formation and wall-detachment mechanisms prevailing in a PEM electrolyser with a spatial resolution of about 250 μm in a time-resolved at 1 Hz.

A number of studies have demonstrated several aspects of the applicability of neutron imaging with reference to microfluidics devices. In an early work by Takenada^52^, the formation of cavitation, i.e. gas bubbles, in a real-size diesel fuel injector was detected, however at a resolution of 30 μm for a 200 μm injector nozzle. More recently liquid/vapour interfaces owing to cavitation in a similar fuel-injection device were visualised with a neutron microscope that can achieve a spatial resolution as low as 5.0 μm for an exposure time of 120 s^53^. Referring to more versatile vortex fluidic devices relevant to applications ranging from chemical-reaction acceleration to nanomaterial fabrication, neutron imaging has been used to measure the thickness of the film forming at the wall of a quartz tube rotating at different speeds up to 9,000 revolutions per minute^54^ with a resolution in the order of 67 μm.

The overview of relevant literature presented underpins the fact that neutron imaging techniques have matured over the last two decades and presently allow the resolution of complex two-phase flow fields in sub-mm length scales. However, the capability to extract quantitative data of vapour at such scales and especially in highly transient flows with phase-change has not been demonstrated in the literature. To the authors’ knowledge, the present work is the first to demonstrate that neutron imaging is suitable for quantifying in-nozzle cavitating flow at the micrometre level, consequently elucidating the distinct forms of vaporous structures that arise. It establishes the relevance of the technique to any flow-mechanics related field where non-intrusive, highly accurate imaging and quantification of interfacial flows is crucial. Given the enhanced spatial resolution achieved, the technique constitutes a potent tool useful for unveiling internal flow processes, apart from the industrial devices already reported, in a versatile range of rapidly-evolving microfluidics and lab-on-a-chip devices, microreactors and diagnostic devices with complex geometrical layouts rendering optical access challenging. In addition, the influence of QAS additives on in-nozzle cavitating flows and spray atomisation has been explored by the authors’ group using different visualisation techniques^22,29,61^. The current study provides solid proof that viscoelasticity modifies the actual composition of the two-phase mixture within the orifice in a quantifiable manner.

## Results

Hydrocarbon fuel samples were selected as working media and examined in a comparative manner, as the flow layout is inspired by fuel injection equipment. A set of flow conditions, corresponding to well-established cavitation within the orifice, have been examined as summarised in Table 1. The non-dimensional numbers shown, namely the cavitation and Reynolds numbers, are indicative of the level of turbulence and the extent of vapour prevailing within the nozzle. Since the metallic needle inserted in the flow chamber upstream the orifice remained static for the presented experiments, two values of lift from the needle seat have been tested (0.5 and 1.0 mm), corresponding to different levels of flow constriction.

### Quantification of in-nozzle vapour generation

**Fig. 1:**
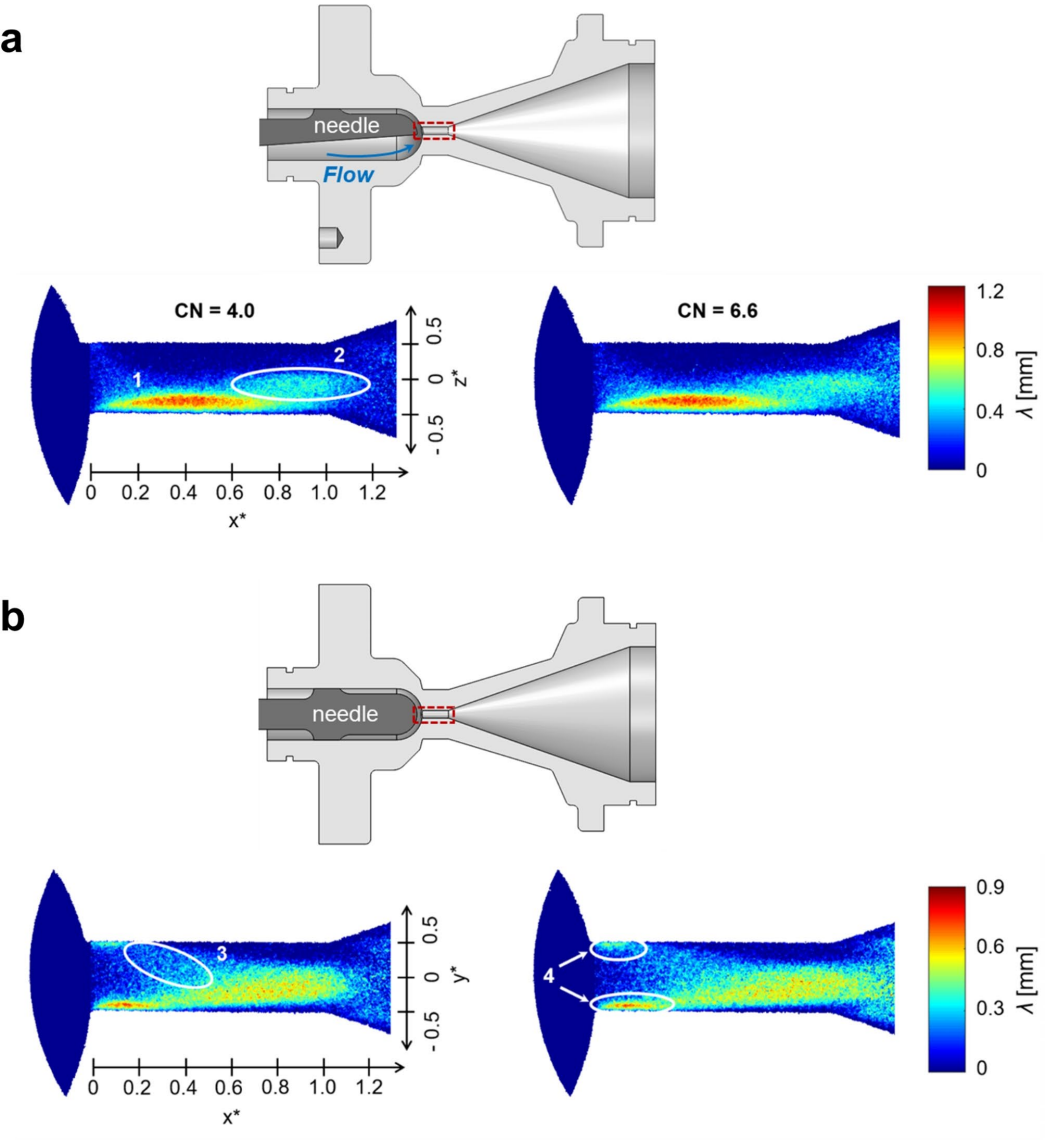
Two-dimensional plots of time-averaged mean vapour-path length along the irradiation line-of-sight, for different values of Cavitation Number (CN), Lift = 1.0 mm: (**a**) side view and (**b**) top view. Both panels refer to reference diesel fuel. The additised sample exhibits qualitatively the same cavitation topology features. The inset of each panel depicts the orifice geometry, where flow is from left to right, while the active window has been annotated as a dashed-line rectangle. Normalised coordinate $\:{x}^{*}$ was defined by dividing the axial coordinate $\:x$ by the orifice length (*L = 5.0 mm*). Likewise, radial coordinates$\:\:{y}^{*}$ and $\:{z}^{*}$ were normalised against the orifice diameter (*d = 1.5 mm*).

Figure 1 depicts two-dimensional plots of the mean vapour-path length along the line of sight for two different orientations of the orifice, conventionally referred to as ‘side’ and ‘top’ views, as explained in the Methodology section. The three-dimensional two-phase flow field emerging in the orifice emanates from the offset of the orifice with respect to the axis of symmetry of the upstream sac region, along with the presence of the needle. The flow is entering mainly from the lower part of the orifice cross-section, as shown on the inset of Fig. 1, hence leading to a three-dimensional flow separation and thus, cavitation pattern. The three-dimensional cavitation topology has also been confirmed by X-ray imaging^33^. Referring to the side view plots, (Fig. 1a), high vapour-path length values can be discerned towards the lower part of the orifice circumference for axial (X) locations in 0 ≤  *x*∗ ≤ 0.8, region (1) indicating the manifestation of an attached cloud cavity, owing to the flow separation induced by the geometric constriction. The plots, although time-averaged representations, provide some insight on the presence of different cavitation regimes within the orifice. For instance, it is worth highlighting the axially elongated region (2) of moderate vapour-path length values of the order of 0.4 mm. The region is located in the vicinity of the orifice axis of symmetry (*z*∗ = 0) and laterally to the trailing edge of the attached cavity. Since the vaporous structures corresponding to region (2) emerge in the orifice core, they can be postulated as transient vortical cavities which form and collapse during the exposure event, thus, resulting to moderate ensemble averaged vapour-path length values. Of course, the knowledge of transient features mainly stems from previous X-ray high-speed imaging performed in a plastic orifice of identical nominal internal flow path^33^.

The top-view plots of Fig. 1b highlight further the presence of vapour structures in the orifice core. Both elongated vortical cavities and shed cloud cavities co-exist in the annotated region (3), which in aggregate, produce vapour-path length values of the order of 0.4 mm or 27% of the orifice diameter. The time-averaged nature of the imaging technique does not allow the quantification of individual transient vortical cavities, hence producing the highly asymmetrical mean vapour-path length distributions of Fig. 1b. The produced results constitute an ensemble average of different structures. Vortical cavitation is common in flow-constricted orifices and is attributed to underlying longitudinal vortices. Such structures set in and collapse with lifetimes of microseconds throughout the exposure time as has been demonstrated in a previous study by the authors^33^ conducted in the same nominal internal geometry. The onset location of vortical cavities is highly sensitive to both operating conditions, e.g., vibrations, and geometrical features, e.g., surface roughness or machining imperfections. Hence, in conventionally-machined orifices vortical cavities do not necessarily appear in the centre of symmetry. The two asymmetrical projections appearing as attached cavities in the top-view plots, region (4), infer that the vaporous structure emerging in the orifice entrance (0 ≤  x* ≤ 0.2), has, in fact, a torus-like shape, with the core being occupied by liquid.

**Fig. 2:**
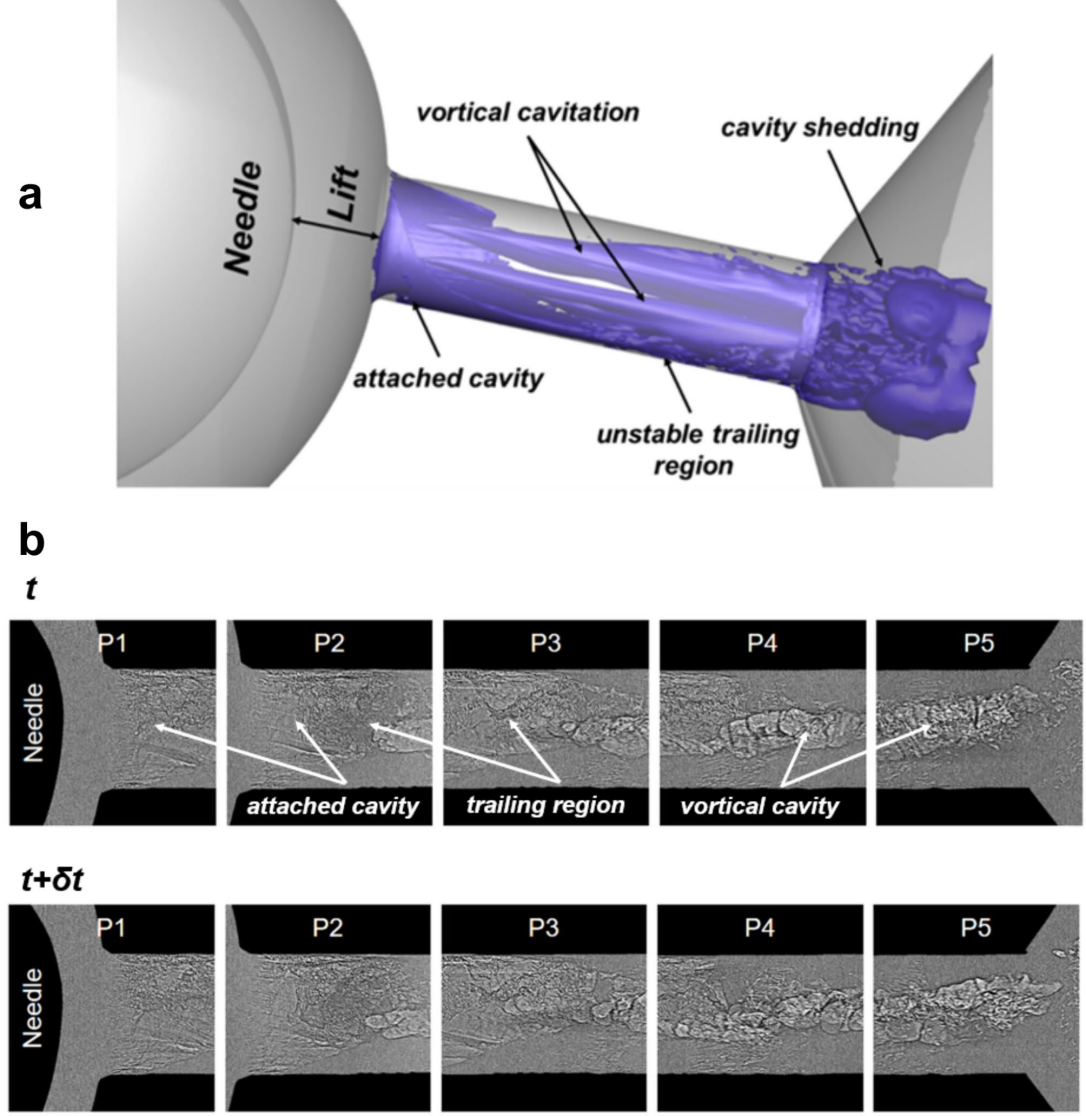
Conceptual three-dimensional cavitation topology within the orifice: (**a**) rendering of prevailing cavitation regimes. Cavitating/vaporous structures correspond to the blue iso-surfaces, while the grey outline corresponds to the orifice internal flow path. (**b**) Time-resolved X-ray phase contrast radiographies obtained in a previous X-ray imaging campaign conducted by the authors’ group^33^ (top view, CN = 6.6, L = 0.5 mm); the full animation is available as Supplementary Material movie SM1. It must be noted that the orifice length of 5.0 mm has been irradiated in a successive manner through overlapping active windows due to 2.5 mm in diameter spot of the X-ray beam. Flow is from left to right in both panels.

The three-dimensional topology of the entire cavitation cloud can be perceived with more clarity by taking advantage of the conceptual cavitation-regime representation of Fig. 2a, produced in a similar flow layout employed for X-ray imaging, along with time-resolved phase-contrast radiographs obtained in a previous campaign^33^ depicted in Fig. 2b. Figure 2a, corresponds to a time instance of the transient two-phase field highlighting the system of prevailing vaporous structures forming within the orifice and in the vicinity of its outlet. Similar to Fig. 1, characteristic cavitation regimes can be discerned, and, in addition, their full three-dimensional topology is revealed. As indicated by the blue iso-surfaces highlighting the prevailing vaporous structures, a toroidal (halo-shaped) attached cavity sets in at the orifice entrance. The two-dimensional projection of this structure corresponds to regions 1 and 4 in the line-of-sight plots of Fig. 1. Instabilities grow at the cavity trailing region, as annotated by the corrugated iso-surface topology and are eventually shed past the nozzle outlet, combined with cloud cavities forming at the outlet region due to the diverging geometry. Cloud cavitation shedding due to geometrical expansion, and consequently flow separation, constitutes a typical transient mechanism, which has been investigated extensively in venturi nozzles^55^. Besides, elongated vortical cavities emerge in the orifice core and occupy the majority of the orifice length, as shown in the conceptual schematic of Fig. 2a and has been verified in previous investigations^28,29,33,56^. In reality, the vortical cavities demonstrate a highly-transient nature, as will be explained in the discussion relevant to Fig. 2b.

The faithful representation of liquid/vapour interfaces by radiographs corresponding to consecutive time instances with a time interval of 14.7 µs provide additional insight on the prevailing cavitation dynamics, as annotated in Fig. 2b. Indeed, the attached cavity can be clearly discerned at the orifice entrance (panel P1), having an unstable trailing region from which vaporous cavities are shed (panels P2, P3). Besides, coherent elongated cavities can also be seen overlapping with the attached cavity for top-view line of sight images (panels P3, P4). The morphology of the vortical cavities is highly transient with the average radius oscillating at a frequency of the order of 1,000 Hz, as demonstrated by the supplementary Movie SM1. Juxtaposition of Fig. 1b (top-view mean vapour-path length) with Fig. 2b and animation SM1, clearly demonstrates that the mean vapour-path length values of the order of 0.3–0.6 mm appearing in locations away from the projected sidewalls correspond to either cavity shedding or longitudinal vortical cavities, as verified by XPCI. It is interesting to notice that phase-contrast imaging is capable of capturing even µm-sized structures in the unstable trailing zone, however, does not offer information on the overall extent of vapour.

**Fig. 3:**
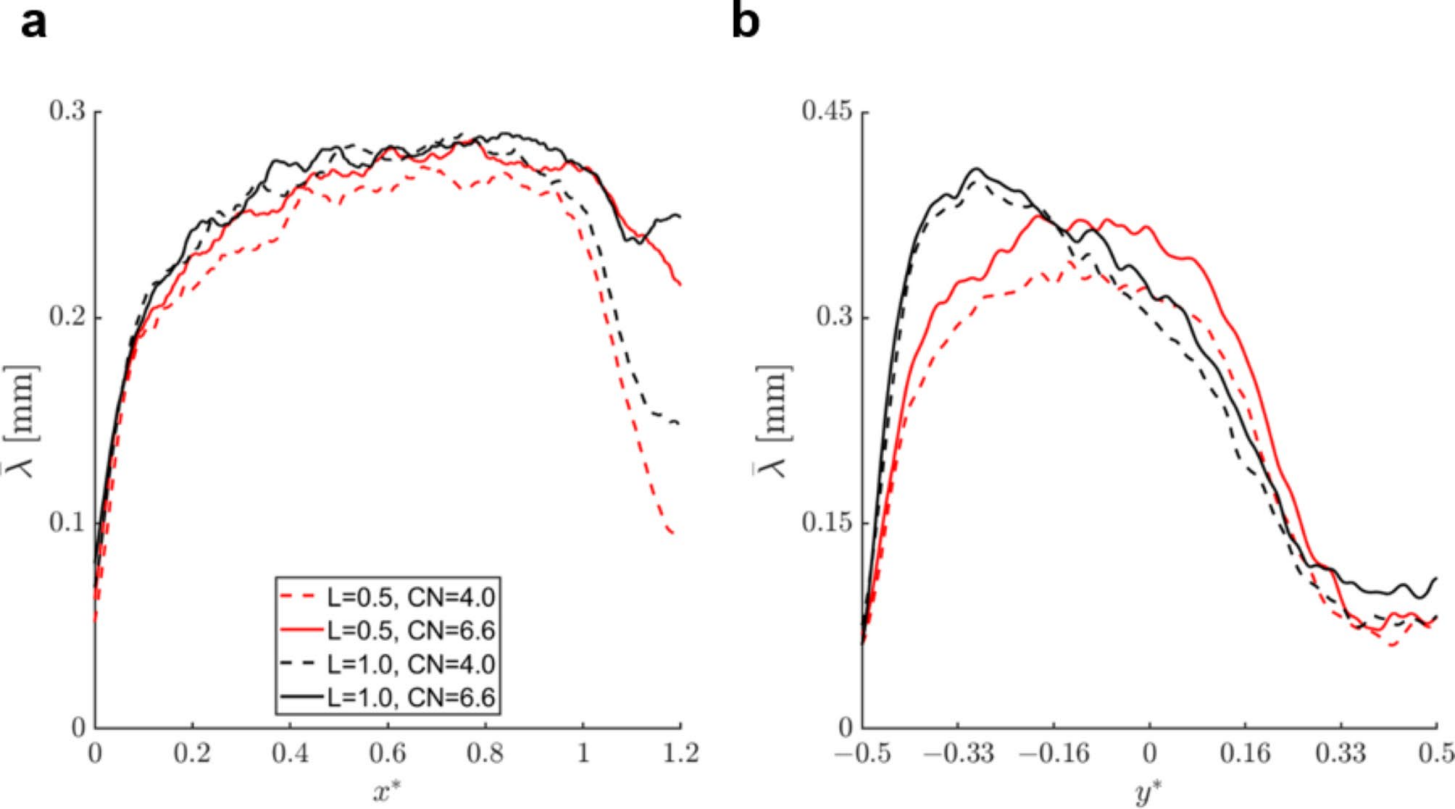
Effect of CN and needle lift on averaged vapour-path thickness distributions attained from top view radiographs of base diesel: distribution of (**a**) axial vapour-path length, (**b**) radial vapour-path length. Each value of the graphs has been produced by line averaging all vapour-path length values along the direction perpendicular to coordinate of the horizontal panel of each panel. The uncertainty associated with the vapour path values lies in the range 0.002–0.015 mm.

Figure 3 illustrates the effect of CN and needle lift on the overall extent and morphology of in-nozzle cavitation through radially- (Fig. 3a) and axially-averaged (Fig. 3b) vapour-path length distributions. It has to be noted that the plotted distributions were derived from the top-view images of Fig. 1b, where the different cavitation regimes and especially the cavity shedding region (annotated as region 3 in Fig. 1b) can be clearly distinguished. Increase of CN from 4.0 to 6.6, has a well expected effect, leading to higher vapour-path length values along both the axial and radial directions. The increase in vapour extent seems to be consistent along both the axial and radial directions. Increase of the needle lift from 0.5 to 1.0 mm does not strongly modify the cavitation topology within the nozzle in a qualitative manner, as similar vaporous structures persist. Nevertheless, clear quantitative differences have been measured with respect to the mean vapour-path length. Firstly, an increase of axial vapour-path length values can be discerned for x^*^> 1 at lift of 1.0 mm, Fig. 3a, suggesting an enhancement of the vapour shedding process at the nozzle diverging section. These findings are in agreement with the conclusions established from the X-ray flow visualisation campaigns also demonstrating that cloud cavitation prevails over the vortical regime for high values of the needle lift^29,33,57^. The radial (axially averaged) distribution of Fig. 3b reveals a clear shift of peak vapour-path values from the orifice core (y^*^= 0) towards the wall, i.e., at − 0.33 < y^*^< −0.16, as the needle lift increases. As shown in Fig. 2b, vortical cavities, constituting structures exclusively filled with vapour, contribute to the vapour fraction (or path length) in regions away from the wall, hence the distribution corresponding to lift of 0.5 mm in Fig. 3b, suggests that vortical cavitation is promoted for low lifts. On the contrary for lift of 1.0 mm, vapour-path values are enhanced at − 0.33 < y^*^< −0.16 suggesting that cloud cavitation becomes the prevalent regime. A similar trend of increasing vapour-path length values at lift of 1.0 mm and CN = 6.6 for cross-flow locations corresponding to 0.33 < y^*^< 0.5 verifies the argument that wall-attached cavitation is enhanced for high lifts as the specific region corresponds to the upper attached-vapour pocket of characteristic region (4), as shown in Fig. 1b.

### Influence of viscoelasticity on different cavitation regimes

**Fig. 4:**
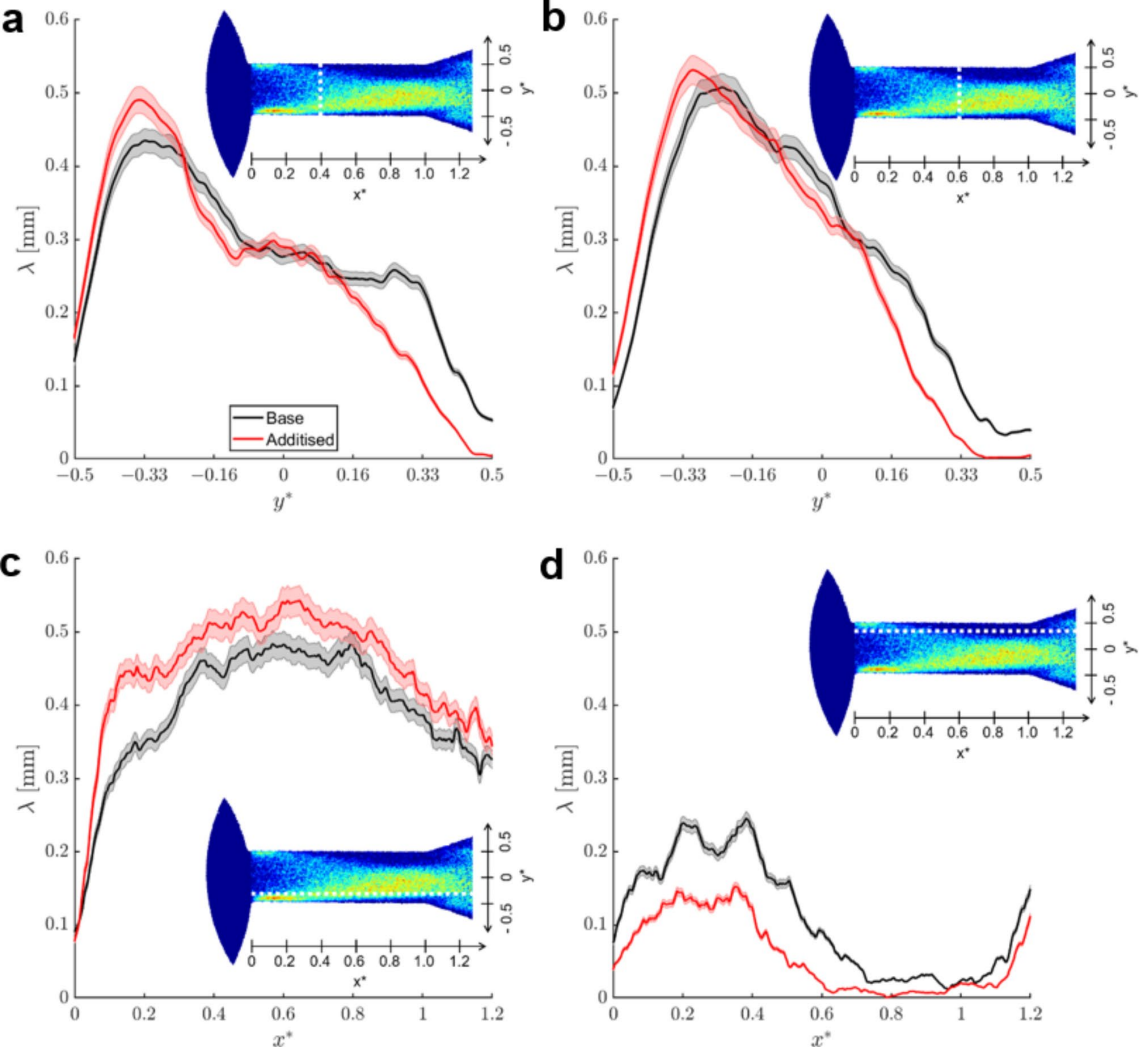
Distribution of mean vapour-path length at characteristic orifice locations, as calculated through the top-view image (CN = 6.6, L = 1.0): (**a**) x^*^ = 0.4, (**b**) x^*^ = 0.6, (**c**) y^*^ = −0.33 and (**d**) y^*^ = 0.33. The locations of the probing lines are shown in the inset of each graph.

Taking advantage of the enhanced spatial resolution of the flow quantification technique, additional test cases were explored, in a comparative manner between the reference liquid and a Quaternary Ammonium Salt (QAS) treated sample exhibiting viscoelastic behaviour. The aim is to underpin subtle differences in the topology of the in-nozzle cavitation that can be attributed to rheological features of the QAS-doped fluid. Figure 4 depicts a comparative assessment between the reference Newtonian sample and the viscoelastic counterpart. Probing lines along the radial direction intersecting the in-nozzle cloud shedding region have been selected to highlight any differences, since previous investigations conducted by the authors’ group have shown that viscoelasticity has a suppressing effect on cloud cavitation^57,58^. Indeed, the distributions at both probing lines, x^*^= 0.4 (Fig. 4a) and x^*^= 0.6 (Fig. 4b) downstream the orifice entrance make clear that the base sample systematically obtains higher vapour-path length values compared to the additised one for y^*^> 0, i.e., at the upper part of the projected orifice circumference, as shown on the inset of each panel. A consistent trend is evident in both probing lines. It is also interesting to notice that for negative y^*^ values, lower than − 0.25, the trend is reversed with the additised sample exhibiting higher vapour-path length values than the base fuel, once again a consistent behaviour in both probing lines. It is not entirely straightforward to pinpoint the underlying cause for the differences observed in the specific regions, which, considering the top-view line of sight, is expected to be occupied by both an attached cavity and transient, elongated vortical cavities, refer also to Fig. 2 and the supplementary movie SM1. Nevertheless, given the fact that wall-attached cavitation corresponds to a relatively thin vapour layer setting in at the nozzle wall, the increased vapour-path length values can be mainly attributed to the more extensive presence of vortical cavities in the region.

Similar conclusions can be established considering the axial distributions of Fig. 4c-d. In agreement with the trends illustrated by the radial vapour-path length profiles, the probing line intersecting the region of the cross-section, at y^*^= −0.33, where vortical cavitation arises (Fig. 4c) clearly shows higher vapour-path length values for the fuel treated with QAS additives in a consistent manner. The respective distribution at y^*^= 0.33 (Fig. 4d), where vortical cavities are completely absent, clearly illustrates an inversed trend. The presence of viscoelastic additives reduces the vapour extent, once again, in a consistent manner throughout the orifice length. In fact, the differences between the two samples are accentuated in the two regions of cloud-shedding, i.e., at 0.2 < x^*^< 0.6 and 1 < x^*^< 1.2, where gradients of the vapour-path length values are evident.

**Fig. 5:**
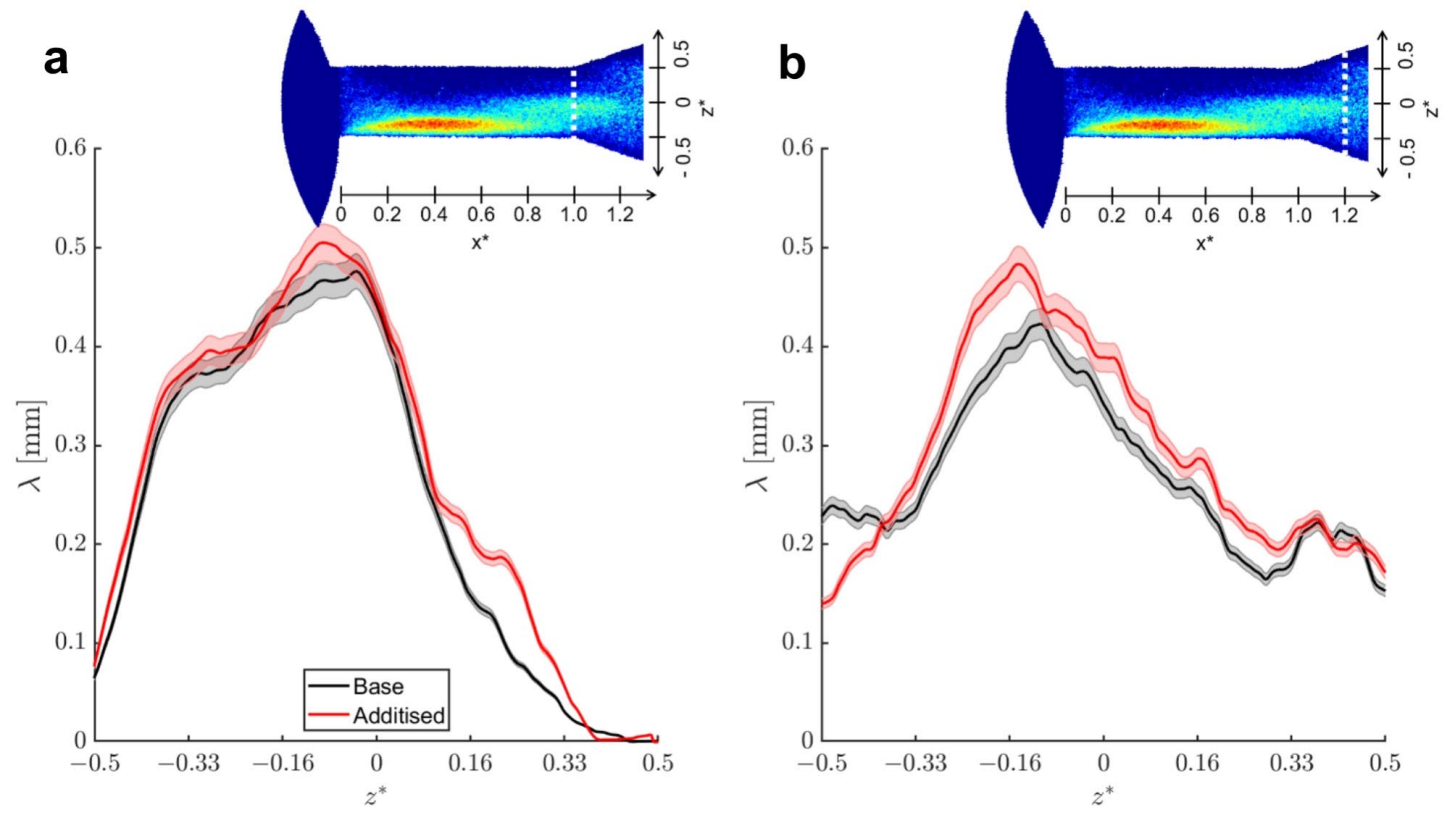
Distribution of mean vapour-path length at characteristic orifice locations, as calculated through the side-view time-averaged image (L = 1.0, CN = 6.6): (**a**) x^*^ = 1.0 and (**b**), x^*^ =1.2. The probing-line locations are shown in the insets of the figure.

To better highlight the enhancement of vortical cavities under the presence of viscoelastic additives, Fig. 5 depicts similar vapour-path length distributions to Fig. 4 yet extracted from side-view images. The contribution of vortical cavitation to vapour-path length values can be discerned with more clarity in the specific line of sight, as its presence becomes evident for larger z-coordinate values compared to the wall-attached regime. Vortical cavities have been postulated to extend past the orifice outlet, i.e., for x^*^> 1.0, refer also to the radiographs of Fig. 2 and the relevant discussion. It is therefore justified by the radial distributions of Fig. 5 to deduce that these vortical cavities, although highly transient in nature and non-static, on average gain in coherence in the case of the additised sample. The radial vapour-path length distribution exactly at the orifice outlet (Fig. 5a) illustrates that the additised sample exhibits consistently higher values than the reference, especially at height between 0.16 < z^*^< 0.33, i.e., above the orifice axis of symmetry. At the specific locations, the presence of vapour can only be associated with vortex-induced cavitation, further strengthening the argument that viscoelasticity has an enhancive influence on vortical cavitation. This trend persists 1.0 mm past the orifice outlet, i.e., 1.0 < x^*^< 1.2, as shown by the probing line at x^*^= 1.2, namely located in the downstream, diverging-flow chamber (Fig. 5b). The vapour-path length values of the additised sample supersede those of the reference in regions away from the wall. Hence, the results are not affected by the flow separation occurring owing to the geometrical expansion and giving rise to cloud-cavity shedding.

**Fig. 6:**
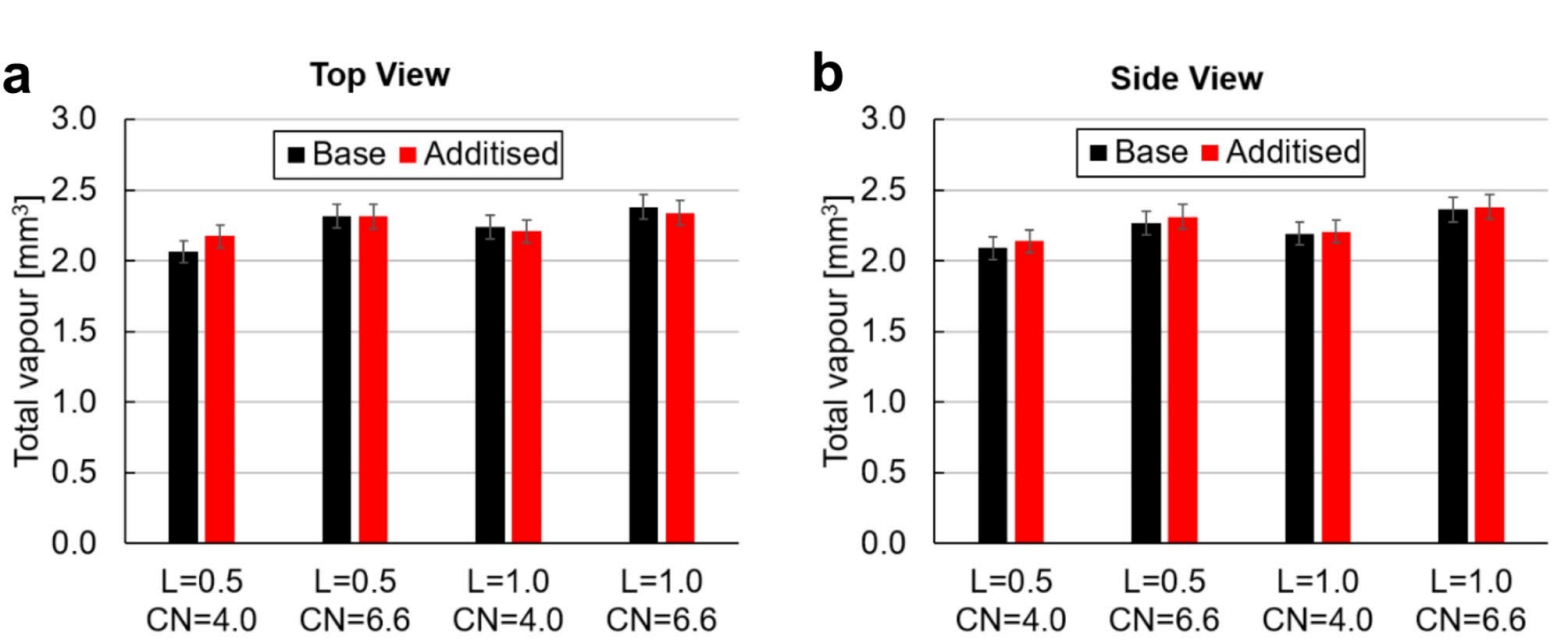
Total vapour volume within the orifice, 0 ≤ x^*^≤ 1.2, for the examined test cases derived by (**a**) top and (**b**) side view images. Error bars encompass the uncertainties in the values of the mean vapour path length and spatial resolution and have been calculated using a typical error propagation methodology^67^.

In order to evaluate if viscoelasticity influences the overall amount of vapour, Fig. 6 presents the total vapour volume calculated as the surface integral of the vapour-path length limited by the nozzle dimensions and the active irradiation window, i.e., 0 < x^*^< 1.2 and − 0.5 < y^*^< 0.5. An initial observation is that, as expected, the overall volume of vapour increases with the cavitation number. Of course, since even for CN = 4.0 cavitation is well established within the nozzle, the influence of wall confinement on flow separation and recirculation leads to a moderate increase in the extent of vapour as CN increases to 6.6. More interestingly, the base and additised samples show comparable values for all cases examined. This indicates that the enhancement of vortical cavitation is counterbalanced by the suppression of cloud cavitation. In other words, the presence of additives does have an overall macroscopic effect, yet it designates the prevailing cavitation regime within the orifice. It is also worth to note that the values obtained by the two lines of sight concur within experimental uncertainty, demonstrating by this way the validity of the quantification method.

## Discussion

### Complementarity to synchrotron imaging

The interaction of X-ray and neutron radiation with matter is characterised by inherent differences in the sense that the former interacts primarily with the electron shell of the atoms/molecules, hence is dependent on the elemental atomic number while the latter is mainly designated by the isotopic composition and, more specifically, by the neutron absorption cross sections of the element isotopes. Subsequently, X-ray irradiation is sensitive to heavy elements, whereas the opposite is true for neutron irradiation. Prior investigations have been conducted by the authors’ group using synchrotron X-ray irradiation^29,33,57^ to elucidate the cavitation topology in opaque orifices of the same internal flow path as that of the current study. Both irradiation techniques are suitable for providing quantitative data provided that the beam energy allows enough signal to be collected on the scintillator aperture and is uniform enough so as not to induce considerable beam hardening effects hindering its accuracy.

**Fig. 7:**
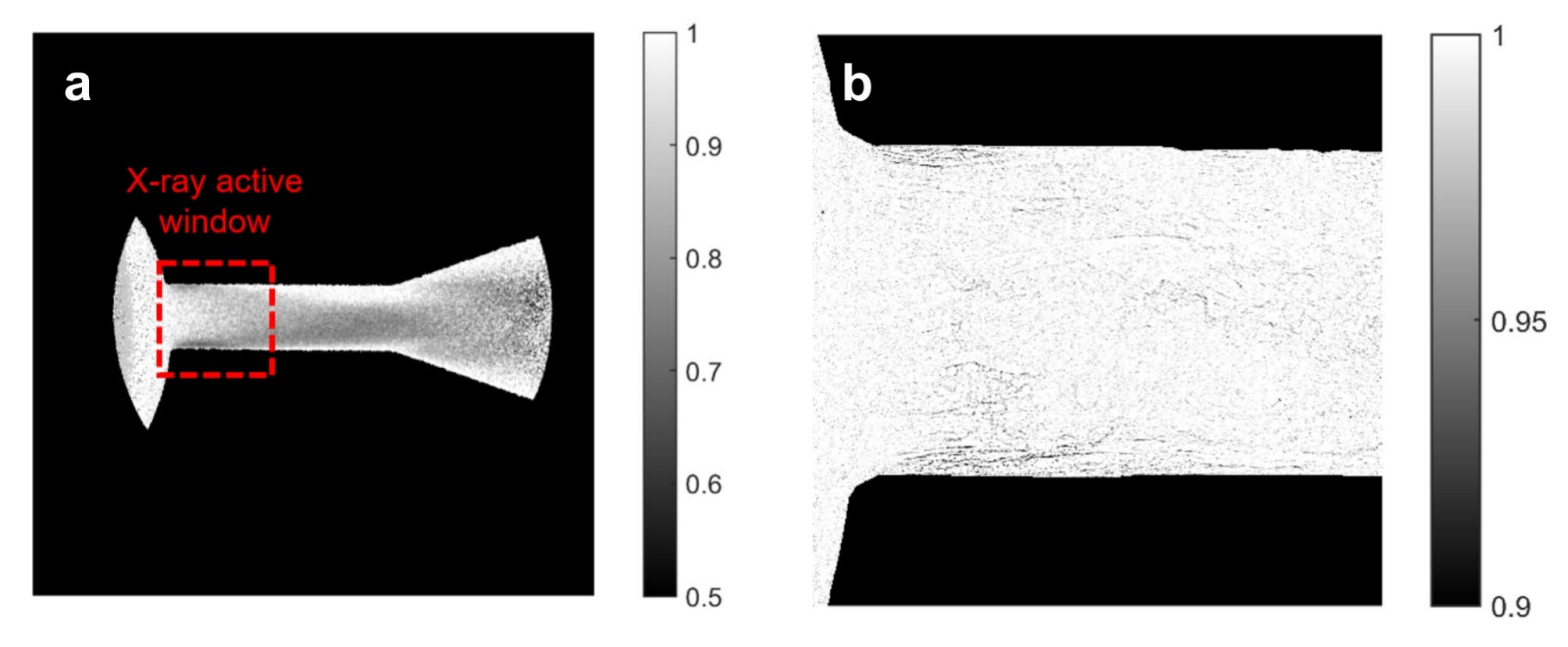
Irradiation transmission $$\:\left(\raisebox{1ex}{${{\rm\:I}}_{stagn}$}\!\left/\:\!\raisebox{-1ex}{${I}_{cav}$}\right.\right)$$ distribution for (**a**) neutron imaging in the present 4-mm thick titanium-alloy orifice and (**b**) X-ray imaging in a 6-mm thick orifice made of polyether ether ketone (PEEK)^33^. The figure depicts top-view images (L = 1, CN = 6.6) of the active window achieved by each method, i.e., 12.9 × 12.9  mm^2^ for neutrons and 2.56 × 2.56 mm^2^ for X-rays. With respect to X-ray imaging, performed employing a ‘pink’ beam with energy of 11.88 keV, the panel covering the inlet section of the orifice has been selected, where the highest vapour-path length values have been detected. Flow is from left to right.

Especially focussing on the visualisation and quantification of wall-bounded cavitating flows, a comparative assessment of past campaigns against the present one allows distinct features of each experimental technique to be identified. In brief, a neutron beam emanating from a spallation source offers a larger imaging spot and higher penetration compared to synchrotron X-rays. Figure 7 depicts, in a comparative manner, the beam attenuation and active visualisation window of the two techniques. It has been verified in the open literature^33,59^ that ‘soft’ X-rays with energies of the order of 12 keV, maximise the contrast in vapour/liquid interfaces in two-phase flows. At such energies, X-rays are incapable of penetrating more than 1–2 mm of common metals. For instance, if 12 keV X-rays were incorporated to irradiate the titanium nozzle examined, the relevant attenuation length (where the beam intensity reduces to $\:1/e$) would be approximately equal to 1.4 mm; however the nozzle thickness is 4.0 mm to be able to withstand internal pressure. Owing to this reason, test-pieces fabricated by organic polymers were incorporated in previous campaigns. However, even in those cases, the contrast obtained by pure X-ray absorption, requiring a beam of narrow energy bandwidth by either using a monochromator or tuning the synchrotron undulator characteristics, is quite weak, refer to the right panel of Fig. 7, and requires a delicate calibration process to be converted to vapour-path length data. On the contrary, the contrast obtained even in uncorrected images acquired by neutron imaging, as shown in the left panel of the Fig. 7, is clearly indicative of the cavitation topology, as revealed by juxtaposition with Fig. 1. This attractive feature of neutron imaging is attributed to the technique sensitivity to light elements.

However, it is important to underpin that synchrotron sources can operate at rapid pulsation modes offering temporally-resolved imaging with frequencies close to 70 kHz and low exposure time of the order of 500 ns, which are suitable for capturing cavitation evolution, suitable for the dimensions and the material of the employed test piece. Frame rates approaching 1 kHz have been reported for time-resolved neutron imaging^60^, hence only time-averaged results can be produced for cavitating flows where vaporous structures evolve in µs time intervals. It is essential to clarify that X-ray imaging of cavitating flow in a steel injector, 200 μm in diameter, has been reported in the literature^27^, however by utilising the full beam spectrum, i.e., a white beam with energy of 50 keV, in order to produce measurable contrast. Hence, the referenced study provided insight on the cavitation topology within the nozzle yet was incapable of producing vapour-path length data. X-ray imaging is primarily oriented towards capturing the dynamic evolution of cavitating structures, while neutron imaging is more suited for quantifying the overall extent of vapour generated in a wall-bounded flow path.

### Influence of fluid rheology on flow topology and phase change

The authors’ group has utilised different imaging techniques to experimentally demonstrate the influence of QAS additives on cavitating flows evolving in injectors and constricted orifices^22,29,61^. In brief, it has been verified that QAS-induced viscoelasticity influences vortical-induced cavitation in an enhancing manner, while the opposite stands for the flow-separation-induced cloud-cavitation regime. The underlying cause has been deduced to be the interaction of polymer micelles with flow recirculation at different scales. Turbulence suppression is a well-known attribute of viscoelasticity^62^, hence the energy of large-scale vortices giving rise to elongated vortical cavitation is dissipated at a smaller rate by turbulent eddies under the presence of viscoelastic additives. On the other hand, wall-attached, cloud cavitation forms in regions of flow separation, where also high shear prevails. Depending on their shape, viscoelastic micelles can form entangled aggregates under high shear, postulated to interfere with the reversed flow at the trailing part of the separated region and, thus, hinder the coherence of the cross-flow recirculation with the same after-effect on the emanating cavity. The detailed mechanism is described in detail by the authors in^29^.

The flow behaviour of the additised sample must therefore be designated by the morphology of the polymeric chains forming due to the QAS macromolecules. Indeed, Small Angle Neutron Scattering (SANS) investigations in a stagnant diesel-fuel surrogate (dodecane) performed in combination with past imaging campaigns^29,57^ have confirmed that for a concentration of 1000 ppm, cylindrical (rod-like) micelles with length and radius of 19.0 nm and 3.5 nm respectively, form in the solvent. Micelles could be detected at concentrations as low as 50 ppm with their length increasing as a function of concentration. The entanglement of cylindrical, or, so called worm-like, micelles as a suppression mechanism for turbulence and cross-flow vortices is therefore justified. The data obtained by neutron imaging corroborate with the conclusions obtained in previous studies and, for the first time, offer quantifiable proof of the influence of the viscoelasticity-inducing agent on the amount of vapour generated within the orifice. It must also be highlighted that the addition of polymers of different chemistry in a hydrocarbon solvent can lead to the formation of micelles of various shapes and sizes. For instance, addition of polyisobutylene succinimide (PIBSI) additives in a hydrocarbon solvent leads to the formation of spherical micelles regardless of the concentration. Proper selection of additive chemistry for the treatment of alternative, non-fossil fuels leading to attractive fuel delivery behaviour, fuel efficiency and reduced emissions constitutes a timely research field for the decarbonisation of the transportation sector beyond passenger cars, namely referring to long-haul vehicles, earth-moving machines, marine vessels, and aircrafts.

## Materials and methods

### Neutron imaging

The spallation neutron source SINQ has a continuous flux of about 10^14^ n/cm^2^/s and offers the possibility to employ low-energy (cold) neutrons to enhance the imaging resolution, since the material macroscopic cross-section is larger for cold compared to thermal neutrons owing to the energy dependency of the absorption cross-section. A higher contrast can therefore be achieved employing cold neutrons, a necessity for two-phase flows to accurately capture liquid/gas interfaces. The neutrons radiating from the ICON beamline are moderated by a liquid D₂ tank at a temperature of 25 K. The neutron beam has an energy equal to 8.53 meV corresponding to a wavelength of 3.1 Å, while the flux of cold neutrons at the beamline is 1.3.10^7^ n/cm^2^/s^63^.

**Fig. 8:**
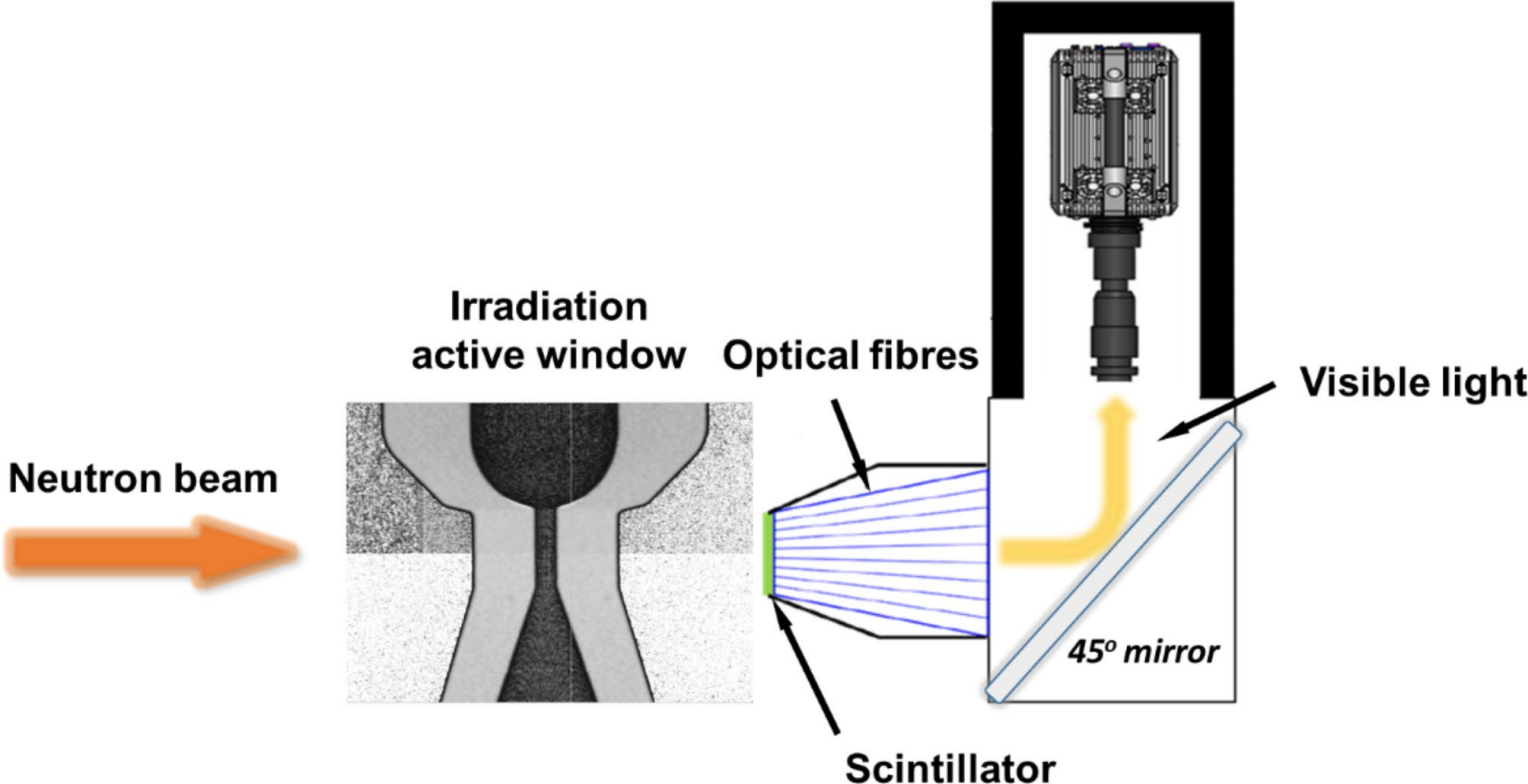
Layout of the irradiation set-up. The FOT intervenes between the scintillator crystal and the 45^o^ mirror inserted to guide the converted visible light to the CMOS camera. The distance between the scintillator frontal surface and the orifice axis of symmetry is equal to 9 mm.

The optical set-up for the visualisation experiments realised the conversion of nuclear radiation to measurable signal through the use of a Gadox-based binder-stabilized scintillator and a CMOS camera. A custom-made Fibre Optic Taper (FOT) device was placed between the scintillator crystal and the camera, as shown in Fig. 8, which enabled the resolution of the detector system to be improved to 16 μm, while resolutions in the range between 30 and 300 μm constitute the current state-of-the-art, as reported in the Introduction section. Minimising the sensor-to-test-object distance and employing a high-resolution scintillator could enhance the resolution further to a value of 10 μm^64^. A FOT comprises a bundle of small boron glass filaments with gradually increasing diameters, while the frontal part of the taper is a cone-shaped piece of glass. Each filament is coated in such a manner so as to guide light travelling within it without any cross-talk with neighbouring filaments. The FOT employed in the experimental campaign has a length of 11 cm and diameters of 14 mm and 78 mm on the scintillator and camera sides, respectively. Its magnification factor is approximately equal to 6^64^. The active window of the optical system was 12.9 × 12.9 mm^2^ and the exposure time to produce time-averaged images was set to 60 s, which was found sufficient to produce well-measurable signal-to-noise ratio. It has to be noted that, with respect to the achievable temporal resolution of neutron irradiation, imaging at 800 fps has been employed for bubbly flows without phase change^65^. However, an order of magnitude higher resolution is required for accurate visualisation of cavitating processes, hence the provision of time-resolved quantitative data is not feasible. The primary aim of the current investigation, therefore, lies on the quantification of the overall vapour extent in a wall-bounded flow layout, demonstrating its applicability to a wide range of bubbly flows, rather than capturing distinct cavitation dynamics.

### Hydraulic loop and instrumentation

**Fig. 9:**
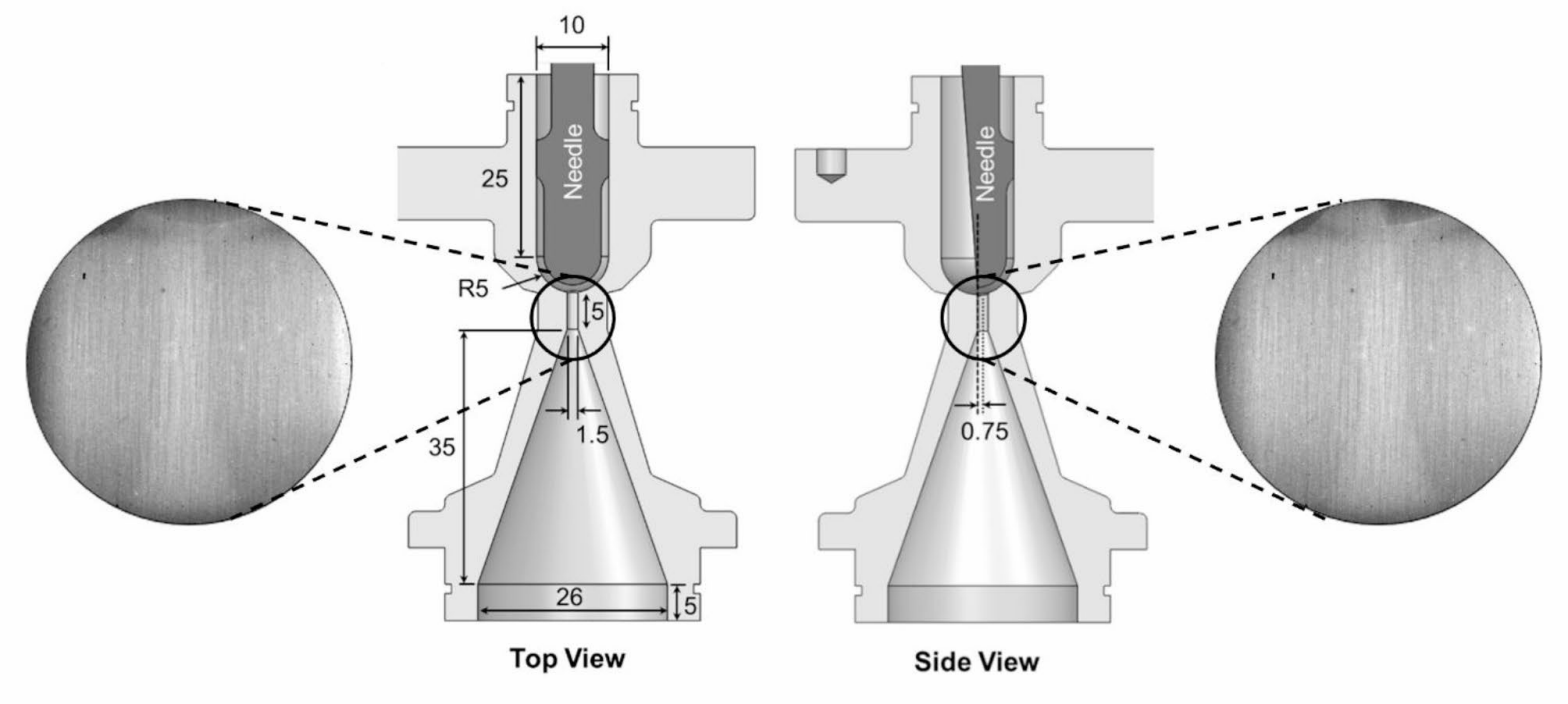
Schematic of orifice employed in the neutron-imaging campaign revealing the internal flow path. The external shape has been modified to allow the scintillator crystal to approach the flow region of interest as much as possible, subsequently enhancing the spatial resolution of the imaging technique. The same nominal flow path has been employed in past campaigns exploring the suitability of synchrotron irradiation for cavitation visualisation and quantification^33^. The wall thickness for the particular test piece is equal to 4 mm. Nevertheless, the orifice manufacturing technique (electrical discharge machining) caused the orifice offset to deviate from the location set in the drawings. Geometry files for the orifice, needle and their assembly (both for 0.5 mm and 1.0 mm lift values) are provided as Supplementary Material to allow the reproduction of the actual test-piece employed in experiments. Dimensions are in mm.

The hydraulic flow loop employed in the experimental investigation comprises a fuel tank, a gear pump to circulate the working medium, and metallic inlet/outlet manifolds connecting the examined test piece with the loop tubing along with flowrate and pressure regulators. Pressure and temperature at the orifice inlet and outlet were constantly monitored with the use of pressure transducers and type K thermocouples, respectively. Flow rate was monitored through an axial-turbine flow meter. The rig has been described in detail in^21^ and was operated under steady-state flow rate conditions for the current experiments. The internal flow path along with basic dimensions of the examined injector-replica orifice are depicted in Fig. 9. As can be seen, an abrupt geometrical constriction is realised between the hemispherical flow-chamber of 5 mm radius and the subsequent orifice of 1.5 mm in diameter. A steel needle with a hemispherical tip is also inserted in the upstream chamber to enhance resemblance to an actual injector device. The needle lift is set using a micrometre fastened to the inlet manifold with a precision of 10 microns. Apart from confirming the micrometre reading for each test case examined, the needle lift position was also verified by direct measurement on the raw images. Apart from confirming the micrometre reading for each test case, the needle lift has also been verified by direct measurement on the raw images The orifice was manufactured from a titanium alloy (Ti-6Al-4 V), which was preferred over iron (steel) alloys due to lower atomic number of pristine titanium. A mild neutron activation was detected past the experiments requiring a three-month quarantine of the test piece. Since a fully three-dimensional cavitation topology was expected to arise within the orifice, the test piece was irradiated at two distinct orientations referred to as ‘top’ and ‘side’ views, refer to the schematic of Fig. 9.

The in-nozzle flow conditions were characterised using the Reynolds and cavitation numbers defined as follows:

1 $$\:Re=\frac{{u}_{ave}\cdot\:d}{{\nu\:}_{fuel}}=\frac{4\dot{V}}{\left(\pi\:d\right)\cdot\:{\nu\:}_{fuel}}$$

2 $$\:CN=\frac{{p}_{inj}-{p}_{back}}{{p}_{back}-{p}_{sat}}$$

where *u*_*ave*_ is the flow average velocity within the orifice, *d* (= 1.5 mm) is the orifice internal diameter, $\:\dot{V}$ is the imposed volumetric flow rate and *ν*_*fuel*_ is diesel kinematic viscosity^17,21,28^ with respect to the definition of the Reynolds number. Flow average velocity was calculated as the volumetric flow rate recorded by the flowmeter divided by the orifice cross sectional area. The values of injection *p*_*inj*_, back (outlet) *p*_*back*_ and saturation pressure *p*_*sat*_ are present in the definition of CN. Liquid thermophysical properties should be considered as those of commercial Diesel blends and can be obtained from open databases^54^, refer also to our previous investigation employing the same fuel samples^61^. It must be emphasised that the base fuel macroscopic properties have been verified to not be affected by the presence of QAS additives (diluted at a concentration of 1000 mg/kg) and therefore matching of the non-dimensional numbers for the two blends examined offers a straightforward comparison. With respect to the rheological properties of the additised fuel sample, it must be pointed out, that its shear relaxation time cannot be reliably measured using state-of-the-art rheometers, owing to the miniscule additive concentration and the low liquid viscosity. However, it is estimated to be of the order of microseconds for a dilute polymer mixture. Fuel temperature was maintained at a temperature of 42.0 ± 1.0^o^C through a water-cooled heat exchanger and a PID controller.

### Image correction and post-processing

The extent of vapour along line of sight was quantified following a post-processing treatment of the raw attenuation images based on attenuation. Presence of vapour within the orifice leads to an increase of the percentage of irradiation $\:{I}^{{\prime\:}}$ reaching the scintillator compared to the respective *I* for the case where the nozzle is filled by liquid. Postulating that beam attenuation occurs only due to absorption in the irradiated material layers, the accumulated vapour thickness can be calculated through the Beer-Lambert law:

3 $$\:\frac{{I}^{{\prime\:}}}{I}=\:\:\:{e}^{-\mu\:\cdot\:\lambda\:}$$

where *µ* is the attenuation coefficient and *λ* is the mean vapour-path length (thickness). The data from which the mean vapour-path length values were calculated constitute an ensemble average of 30 independent irradiation events for the same operating conditions with each event having an exposure time of 60 s.

In order to be able to obtain quantitative values for the mean vapour-path length λ that correspond exclusively to irradiation absorption, raw images must be normalised against background images of stagnant flow, i.e., with pure liquid in the orifice and corrected, in terms of signal or pixel-brightness values, for beam irradiation distribution, irradiation scattering as well as camera dark current. Hence, with respect to correction procedures, open-beam images were obtained by removing the sample from the beam path, while dark-current images were obtained in the absence of any illumination, in essence measuring background noise and camera offset.

**Fig. 10:**
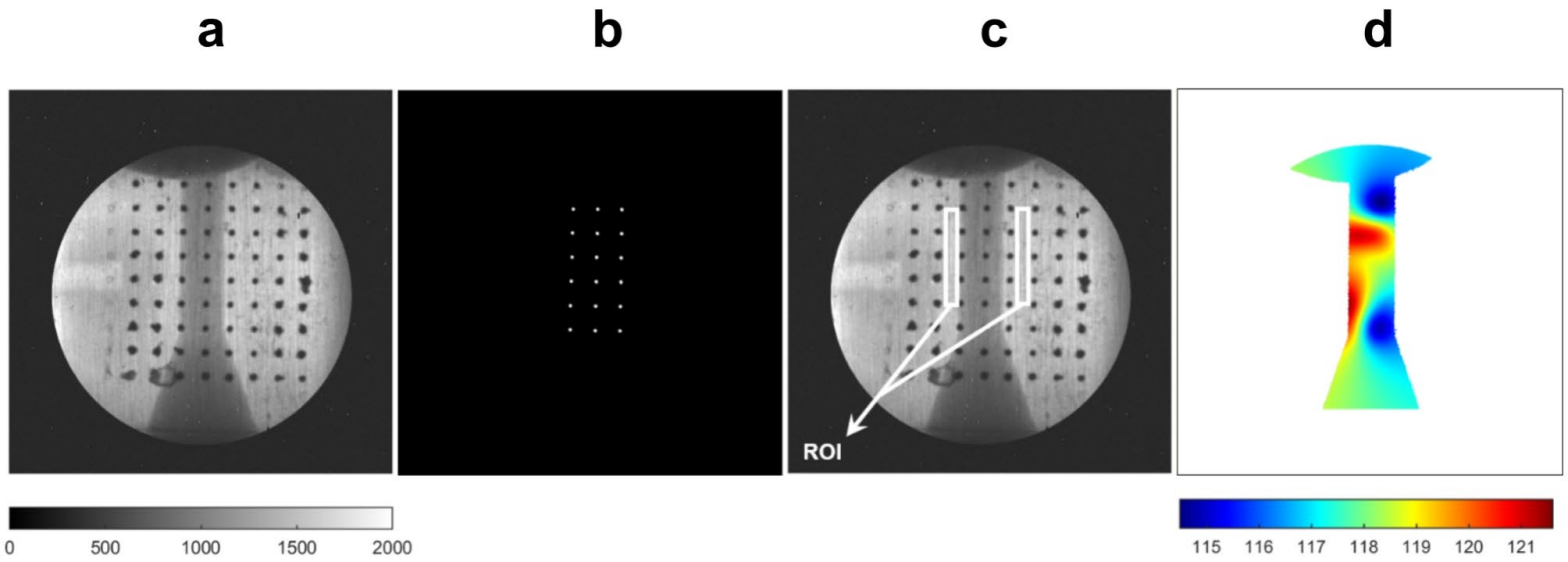
Procedure to correct for scattering effects (Side view, base diesel, CN = 6.6, L = 1 mm): (**a**) raw image with matrix of BBs present, (**b**) post-processed mask image, (**c**) regions of interest for the calculation of the dose operator and (**d**) correction map to account for scattering effects. The colour band range is set accordingly to accentuate scattering effects and enhance panel clarity. As a reference, the irradiation intensity in the stagnant flow within the orifice is of the order of 1100 counts.

To determine the sample and background scattering components, two additional irradiation events were performed with the test object either intercepting the beam or not. However, in both cases, images were acquired with a metallic frame containing perfect neutron absorbers or Black Bodies (BB) placed in between the beam and the scintillator, refer to Fig. 10. Since BBs are opaque to neutrons, the measured signal at scintillator locations opposing them corresponds exclusively to the respective neutron scattering component. In other words, a modified attenuation law employed to produce mean vapour-path length *λ* values is as follows:

4 $$\:\frac{{I}_{stagn}-{I}_{scatter,stagn}-{I}_{DC}}{{I}_{cav}-{I}_{scatter,cav}-{I}_{DC}}=\:{e}^{-\mu\:\lambda\:\:}$$

where *I* corresponds to the contrast count per pixel of flat-field corrected images to account for variations in the beam intensity. From an image post-processing perspective, in order to calculate the contribution of scattering, locations of BBs were detected on the raw images and circles with a radius of a third of the physical radius were drawn to create a mask, refer to Fig. 10a-b. Applying this mask-image on a dark-current corrected image of either stagnant liquid or cavitating flow allows the determination of the scattering contribution in terms of brightness counts. Values acquired in the BB locations were subsequently interpolated throughout the whole image using a discrete Laplacian over the 2D region and solving the Dirichlet boundary condition problem.

It is important to note that the presence of the BBs themselves influences the overall neutron flux reaching the scintillator. Therefore a, so called, dose operator D in accordance with practices for neutron-imaging quantification^66^ was calculated in regions outside the orifice active region excluding BB locations, as annotated in the regions of interest of Fig. 10c The dose operator was calculated using the dark-current corrected stagnant-flow images, where the averaged (within the ROI) intensity in the absence BBs was divided by the equivalent one for BBs present, as follows:

5 $$\:D={\left(\frac{{I}_{stagn}-{I}_{DC}}{{I}_{{stagn}_{BB}}-{I}_{DC}}\right)}_{ROI}$$

The resulting map of correction values (Fig. 10d) results by multiplying the dose operator by the 2D matrix of interpolated scattering-contribution values and is applied on a per-pixel manner to raw images to account for scattering effects in the attenuation law of Eq. (4). It should be noted that the probability of a neutron scattering event is characterised by cross-sections, i.e., essentially probabilistic measures of the likelihood of a particular interaction (scattering or absorption) occurring when a neutron encounters a nucleus. Hence, neutron scattering is intrinsically a probabilistic event and the relevant scattering correction matrix has been formulated for each test case examined.

**Fig. 11:**
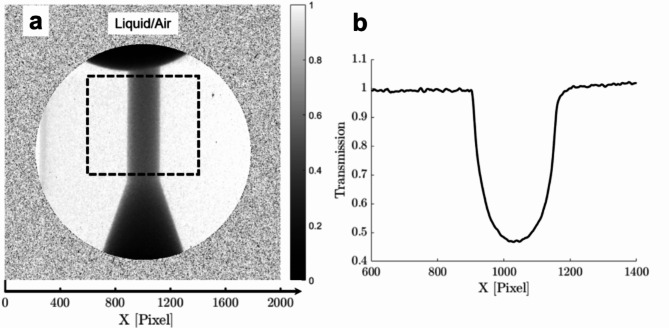
Calibration process for the determination of the absorption coefficient: (**a**) Contour plot of transmission derived by dividing irradiation images of the orifice occupied by stagnant liquid and air. The annotated window corresponds to the region considered for the determination of the absorption coefficient. (**b**) Average transmission distribution within the sampling window. The part corresponding to the exponential decay in the middle part of the distribution (fluid region) has been utilised to derive the absorption coefficient, while the fringes evident on either side that corresponds to the metallic part of the test piece are indicative of scattering effects.

Absolute values of the mean vapour-path length along the line of sight can only be obtained if the absorption coefficient *µ* of Eq. (4) is estimated through an appropriate calibration process. Radiographs of the orifice filled either with stagnant liquid or air (in essence having a density representative of diesel vapour) were obtained and *µ* was calculated employing the attenuation law as follows:

6 $$\:T=\frac{{I}_{liquid}}{{I}_{air}}={e}^{-\mu\:\cdot\:d}$$

where the transmission T for the calibration case resulted as the contrast ratio between the air- and liquid-filled orifice and *d* is the cross-flow orifice chord (i.e., along the line of sight) at any given pixel. Transmission *T* was averaged in 600 rows covering the orifice length, refer to Fig. 11, and fitted with an exponential curve to produce a value for the absorption coefficient equal to 0.512 ± 0.002.

### Experimental uncertainty

A partial derivative error propagation analysis, as outlined in^67^ has been applied to assess the uncertainties in the physical quantities calculated based on primary measurements. With respect to the non-dimensional number characterising the flow conditions, the maximum experimental uncertainty associated with the Reynolds and Cavitation numbers were 3.1% and 1.7%, respectively, based on instrumentation accuracy and geometrical tolerances.

The uncertainty in the mean vapour-path length values, *ε*_*λ*_ is based on the propagation of errors in the derivation of transmission *T* and the absorption coefficient *µ*, as follows:

7 $$\:\frac{{\epsilon\:}_{\lambda\:}}{\lambda\:}={\left[{\left(\frac{{\epsilon\:}_{\text{l}\text{n}\left(T\right)}}{\text{l}\text{n}\left(T\right)}\right)}^{2}+{\left(\frac{{\epsilon\:}_{\mu\:}}{\mu\:}\right)}^{2}\right]}^{0.5}={\left[{\left(\frac{{\epsilon\:}_{T}/T}{\text{l}\text{n}\left(T\right)}\right)}^{2}+{\left(\frac{{\epsilon\:}_{\mu\:}}{\mu\:}\right)}^{2}\right]}^{0.5}$$

The uncertainty in transmission *ε*_*Τ*_ was obtained considering the deviation of the measured transmission values against the theoretical ones, as predicted by Eq. (3). Errors associated with the dark-current and scattering corrections were deemed as non-critical for the attenuation ratio designating transmission and were, thus, omitted. In addition, the uncertainty associated with the absorption coefficient *µ* is estimated following the rationale of Eq. (8), since $$\:\mu\:=-ln\left(T\right)/d$$, where $$\:T=\raisebox{1ex}{${I}_{liquid}$}\!\left/\:\!\raisebox{-1ex}{${I}_{air}$}\right.$$ and *d* is the orifice diameter, as visualised in the calibration test. The uncertainty in the orifice diameter due to manufacturing imprecision and imaging spatial resolution account to 0.4%, and eventually propagates to the overall error associated with $$\:{\epsilon\:}_{\mu\:}$$ values, which is equal 2.2%. The transmission values in all examined cases lie in the range 0.45–1.0 and the average uncertainty $$\:{\epsilon\:}_{\lambda\:}$$ is determined to be of the order of 3.7%. For more details on the uncertainty analysis, please refer to^33^.

**Table 1:** Matrix of experimental test cases. The same flow conditions and needle lift have been applied for the two diesel blends examined, namely a conventional multi-component diesel sample, and a non-Newtonian counterpart treated with Quaternary Ammonium Salt (QAS) additives. The last two columns of the table correspond to the pressures measured at the orifice inlet (p_inj_) and outlet (p_back_), respectively. CN values reveal that well-established cavitation conditions should be expected within the orifice.

Case no.	Needle lift [mm]	Re	CN	*P*_inj_∙10^5^ [Pa]	*P*_back_∙10^5^ [Pa]
1	0.5	31,000	4.0	35.6	7.2
2			6.6	35.1	4.8
3	1.0	31,000	4.0	34.4	7.0
4			6.6	34.9	4.7

## Electronic supplementary material

Below is the link to the electronic supplementary material.

Supplementary Material 1

Supplementary Material 2

Supplementary Material 3

Supplementary Material 4

Supplementary Material 5

Supplementary Material 6

## Data Availability

The datasets generated and analysed during the current study are available from the corresponding author on reasonable request.
