# Supplementary material for: Quantification of cavitating flows with neutron imaging
Source: Sci Rep. 2024 Nov 6;14:26911. doi: 10.1038/s41598-024-76588-3 (PMC11541883; doi:10.1038/s41598-024-76588-3)
Supplement: Supplementary file 6 — Supplementary Material 6 [file 41598_2024_76588_MOESM6_ESM.docx]

**Video 1:**  X-ray phase-contrast radiographies obtained with a temporal resolution of 67,890 Hz (top view, CN=6.6, L=0.5 mm).
